# Isolated Renal Calyceal Urothelial Carcinoma Effectively Treated With PD-1 Inhibitor Alone: A Case Report And Literature Review

**DOI:** 10.3389/fonc.2022.866013

**Published:** 2022-05-10

**Authors:** Shihao Li, Yi Zhu, Zhijian Xu, Jianjun Liu, Hongwei Liu

**Affiliations:** Department of Urology, Affiliated Hospital of Guangdong Medical University, Zhanjiang, China

**Keywords:** immunotherapy, programmed cell death protein-1 inhibitor, tislelizumab, isolated kidney, urothelial carcinoma

## Abstract

The discovery of immune checkpoint inhibitors (ICIs) represents a significant step forward in the battle against malignant tumors. In a number of advanced malignancies, ICIs, such as antibodies to programmed cell death protein-1 inhibitor (PD-1) and its ligand, programmed death-ligand 1 (PD-L1), have shown good therapeutic benefits. A 71-year-old male patient was diagnosed with solitary renal calyceal urothelial cancer. The space-occupying lesion in the upper calyx of the left kidney dramatically decreased after 13 treatments with a PD-1 inhibitor (tislelizumab) alone, and the tumor reached partial remission. This case suggests that a PD-1 inhibitor (tislelizumab) alone may be an effective treatment strategy for solitary renal calyceal urothelial carcinoma.

## Background

Urothelial carcinoma (UC) is the most frequent tumor of the urinary system and is a multisource malignant tumor arising from the urothelium. It includes renal pelvic cancer, urethral cancer, bladder cancer, and ureteral cancer. Most UC is non-muscle invasive, with approximately 30-40% of UC being muscle-invasive or metastatic (1). Among these, urothelial carcinoma of the upper urinary tract (UTUC) is relatively uncommon, accounting for approximately 5-10% of UC cases, with a population incidence of approximately 0.002% (2). The standard therapy for UTUC without metastasis is still radical nephroureterectomy. However, treating solitary renal calyceal urothelial carcinoma remains a significant issue for doctors worldwide.

Immune checkpoint inhibitors (ICIs), such as antibodies to programmed cell death protein-1 inhibitor (PD-1) and its ligand, programmed death-ligand 1 (PD-L1), have made significant advances in the treatment of many tumors, such as metastatic urothelial carcinoma (mUC) in recent years (3). PD-1 receptors are found on the cell surface. When PD-L1 binds to PD-1, it can block the functions of T cells, resulting in downregulation of the immune system and self-tolerance (4). There is still no recognized biomarker that can predict response to ICIs. A recent meta-analysis showed that PD-L1–positive mUC patients who were received ICIs had a better survival benefit than those who were received standard chemotherapy. However, no benefit was observed in PD-L1–negative mUC patients (5). Tislelizumab is a monoclonal antibody directed against PD-1, and was approved in China in April 2020 for patients with locally advanced or metastatic urothelial cancer who have previously received treatment. By lowering its binding to FcγR on macrophages, it can abolish antibody-dependent phagocytosis (6). Tislelizumab was found to have good antitumor effectiveness and tolerability in a range of solid tumors in a clinical trial in China, including non-small-cell lung cancer, melanoma, esophageal cancer, and others (7). We present the case of a 71-year-old man who had isolated renal calyceal urothelial cancer. The space-occupying lesion in the upper calyx of the left kidney dramatically decreased after 13 rounds of treatment with a PD-1 inhibitor (tislelizumab) alone, and the tumor reached partial remission.

## Case Presentation

**Figure 1:**
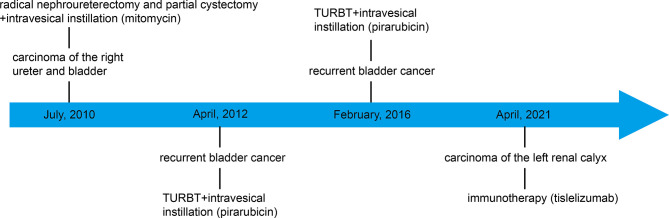
Timeline of the case history.

**Figure 2:**
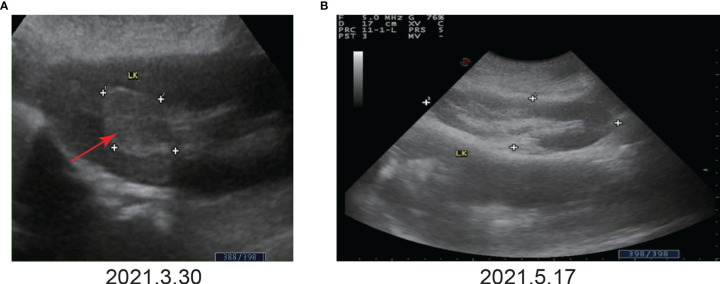
Color Doppler ultrasound before and after immunotherapy. **(A)** Before immunotherapy. Color Doppler ultrasound showed a space-occupying lesion in the upper calyx of the left kidney, approximately 33 mm×24 mm. **(B)** After the third immunotherapy. Color Doppler ultrasound showed that no tumor was found.

**Figure 3:**
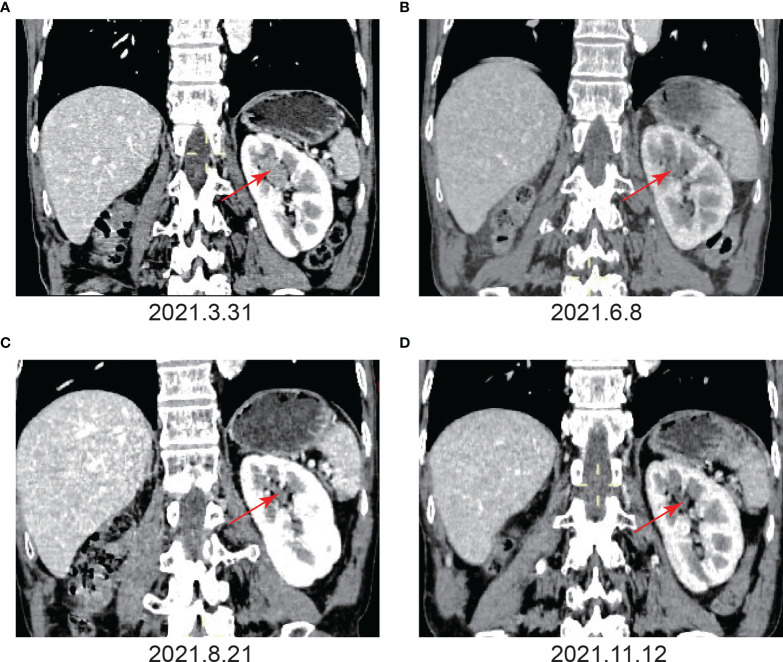
Imaging examination before and after immunotherapy. **(A)** Before immunotherapy. Enhanced CT showed the absence of the right kidney, a space-occupying lesion in the upper calyx of the left kidney, approximately 27 mm×22 mm×13 mm, uniform density, partly with the renal medulla unclearly, and the enhanced scan showed obvious continuous enhancement. **(B)** After the fourth immunotherapy. The space-occupying lesion in the upper calyx of the left kidney was significantly smaller than before, and the size was approximately 12 mm×10 mm. **(C)** After the seventh immunotherapy. The space-occupying lesion in the upper calyx of the left kidney had shrunk to 4 mm×4 mm, and the enhancement degree of the lesion was significantly lower than before. **(D)** After the ninth immunotherapy. The space-occupying lesion in the upper calyx of the left kidney had shrunk to 3 mm×4 mm.

On March 29, 2021, a 71-year-old man presented at the hospital after three days of painless gross hematuria. A 33 mm×24 mm space-occupying lesion of the left renal calyx was discovered using color Doppler ultrasound (March 30, 2021) **(** **Figures 1**, **2A** **)**. In 2010, the patient underwent radical nephroureterectomy and partial cystectomy for carcinoma of the right ureter and bladder. After the operation, mitomycin at a dosage of 20 mg was used for regular intravesical instillation. Bladder cancer recurred in 2012 and 2016, with transurethral resection of bladder tumor (TURBT) conducted in 2012 and 2016. The first pathological investigation indicated that the patient had low-grade noninvasive papillary urothelial cancer **(** **Supplementary Figure 1A** **)**. The urothelial carcinoma of the bladder had penetrated into the lamina propria, according to the second pathological investigation **(** **Supplementary Figure 1B** **)**. After the operations, pirarubicin at a dosage of 30 mg was used for regular intravesical instillation. Abdominal enhanced computed tomography (CT) and urine cytology was performed to further corroborate the diagnosis. Abdominal CT (March 31, 2021) revealed the loss of the right kidney as well as the presence of a space-occupying lesion in the left renal calyx. The space-occupying lesion in the left renal calyx was approximately 27 mm×22 mm×13 mm in size, uniform density, partly with the renal medulla. The plain scan CT value was approximately 35 HU, and the contrast CT demonstrated evident continuous enhancement **(** **Figure 3A** **)**. Three urine cytology results indicated a propensity for low-grade atypical urothelial cells **(** **Supplementary Figure 2A** **)**. Combined with the patient’s clinical symptoms and auxiliary examinations, the initial diagnosis was carcinoma of the left renal calyx.

Given that the patient only had one kidney, there was potential for long-term dialysis or renal transplantation post-surgery. As a result, the patient refused surgery. In addition, the patient refused chemotherapy due to the possible effects of cisplatin-based chemotherapy on renal function. Hence, the patient was treated with tislelizumab alone at a dosage of 200 mg every three weeks. The patient’s color Doppler ultrasound was reexamined after the third immunotherapy (May 17, 2021), and no tumor was found **(** **Figure 2B** **)**. Following the fourth immunotherapy (June 08, 2021), computed tomography urography (CTU) revealed the space-occupying lesion in the upper calyx of the left kidney was substantially smaller than before, measuring approximately 12 mm×10 mm **(** **Figure 3B** **)**. Two urine cytology results indicated atypical urothelial cells, whereas one was negative **(** **Supplementary Figure 2B** **)**. Tislelizumab was regarded as effective since the space-occupying lesion in the upper calyx of the left kidney greatly decreased following treatment. As a result, the patient was treated in accordance with the initial treatment plan. We refined the CTU once again to analyze the efficacy of immunotherapy when the patient was treated with the seventh immunotherapy (August 21, 2021). The CTU revealed that the lesion in the left kidney had reduced to 4 mm×4 mm, and the degree of enhancement had decreased markedly **(** **Figure 3C** **)**. We performed CTU and color Doppler ultrasound when the patient was treated with the ninth immunotherapy (November 12, 2021). CTU revealed that the space-occupying lesion in the upper calyx of the left kidney had decreased to 3 mm×4 mm **(** **Figure 3D** **)**, and color Doppler ultrasound revealed no lesion.

**Figure 4:**
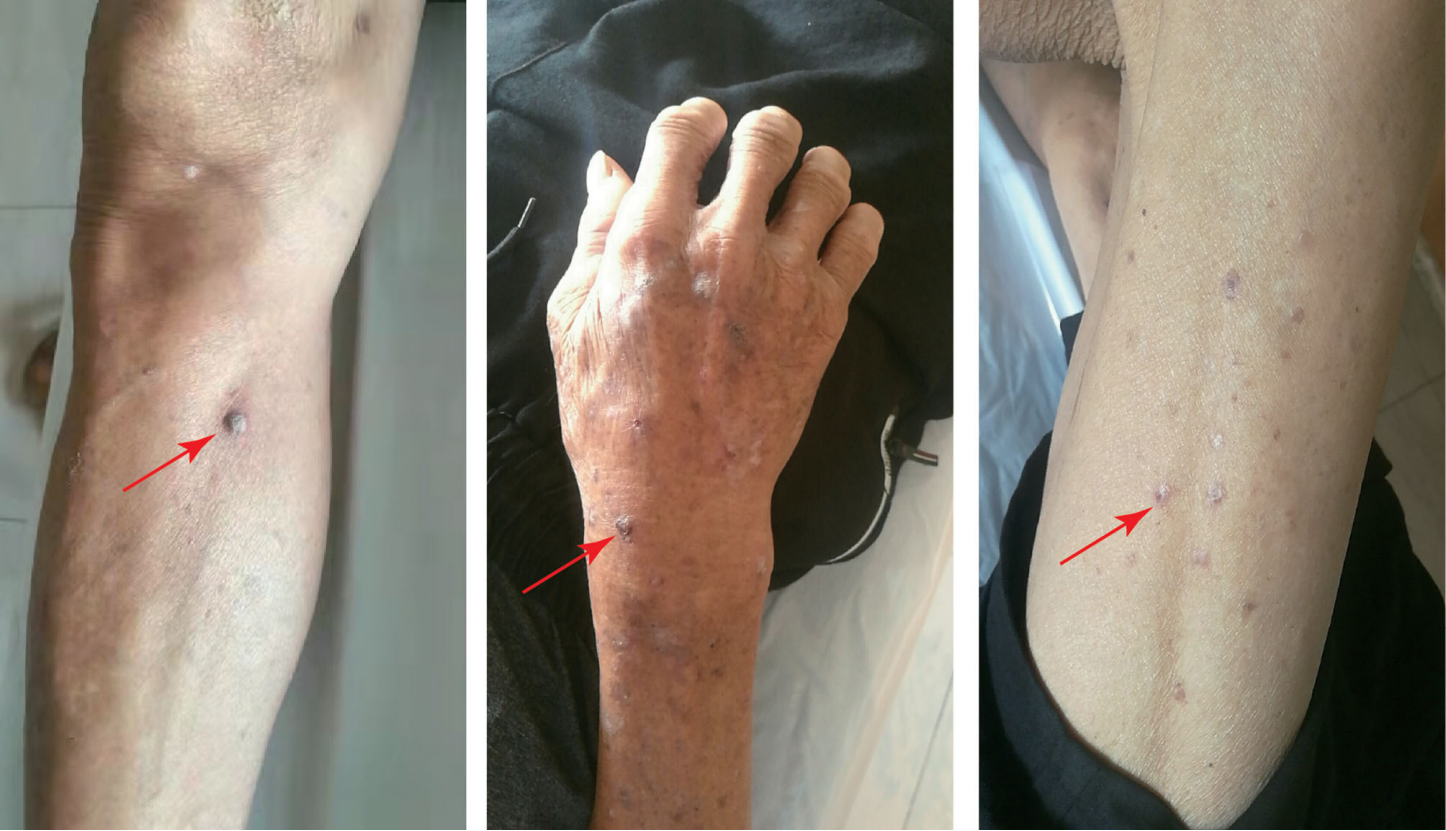
The patient experienced mild cutaneous responses. Scattered gray papules formed on the skin of the patient’s limb.

To date, the patient has been treated with immunotherapy 13 times, and the patient has survived without progression for more than nine months. The patient experienced only a mild cutaneous response. Scattered gray papules formed on the skin of the patient’s limb **(** **Figure 4** **)**. The laboratory examination, which included routine blood work, liver function, and kidney function tests, revealed no evident abnormalities, suggesting that tislelizumab was well tolerated by the patient. Next, we consider continuing to use the original plan to treat the patient, hoping to achieve complete remission of the tumor.

## Discussion

UTUC is a highly invasive tumor, accounting for approximately 5%-10% of UC (2). Although radical nephroureterectomy can achieve a good prognosis, the treatment of UTUC still faces great challenges to some patients who cannot tolerate surgery or chemotherapy, especially for patients with isolated kidneys. ICIs have been shown to be effective in treating a number of malignant tumors, including lung cancer, Hodgkin’s lymphoma, UC, and melanoma (7, 8). Nevertheless, ICIs are not effective against all cancers. The total response rate of PD1/PD-L1 inhibitors in solid tumors is approximately 20%-40% (9).

As an immune checkpoint receptor, PD-1 is mostly expressed in T cells, monocytes, and NK cells, but its major ligand, PD-L1, is primarily found on the surface of dendritic cells and macrophages (10). PD-L1 can, however, be found on the surfaces of tumor cells (10). When PD-L1 binds to PD-1, it can inhibit the activity of T cells, leading to downregulation of the immune system and self-tolerance (4). This is because certain tumor cells express PD-L1, which suppresses T cells’ antitumor function, resulting in the development of tumors (11). Although radical nephroureterectomy is the conventional treatment for nonmetastatic UTUC, long-term dialysis or renal transplantation might have been necessary with this patient because they have only one kidney. In addition, cisplatin-based chemotherapy may affect renal function. Following extensive communication with the patient, the patient declined surgery or chemotherapy. According to the patient’s wishes, after signing the informed consent form, we directly started the second-line treatment with immunosuppressants.

The Food and Drug Administration (FDA) has authorized three PD-1 and two PD-L1 inhibitors, mostly for patients whose malignancies advanced during or after platinum-based treatment. Tislelizumab was approved in China in April 2020 for patients with locally advanced or metastatic urothelial cancers who have previously received platinum-based treatment. Tislelizumab is a monoclonal antibody against PD-1 that reduces antibody-dependent phagocytosis by inhibiting its binding to FcγR on macrophages (6). Tislelizumab exhibits a greater affinity for PD-1 than pembrolizumab and nivolumab, which may be related to tislelizumab’s different binding orientation to PD-1 (7). The off-rate of tislelizumab is 100 times slower than that of pembrolizumab and 50 times slower than that of nivolumab (7). The goal of a trial in China (CTR20160872) is to assess the antitumor efficacy, safety, and tolerability of tislelizumab in adult patients in China who have advanced solid neoplasms. Tislelizumab was administered intravenously to 300 patients with advanced solid tumors at a dosage of 200 mg every three weeks, with a median follow-up period of 8.1 months. The results revealed that 18% of the 251 individuals whose efficacy could be evaluated attained clinical remission. Furthermore, the majority of the adverse events were Grade 1 or Grade 2, with anemia and increased aspartate aminotransferase being the most prevalent, demonstrating that tislelizumab has a good antitumor impact in a range of solid tumors and is well tolerated in the population (7). Tislelizumab was evaluated in a single-arm phase 2 study (NCT04004221/CTR20170071) to determine whether it is safe and effective in patients with PD-L1-positive urothelial cancer. There were 113 individuals in the experiment, with a median follow-up period of 9.4 months. Only 104 patients can be used to evaluate the efficacy, and the objective remission rate is 24%, with 9.6% of patients experiencing full remission and 14.4% experiencing partial remission (6). The case reported in this article achieved partial remission after 13 administrations of tislelizumab. Only mild cutaneous adverse reactions occurred during therapy, and laboratory examinations revealed no evident abnormalities, showing that tislelizumab was safe, efficacious, and well-tolerated in this patient. There are many reports of effective therapy of advanced urothelial carcinoma with a PD-L1 inhibitor alone. Wang et al. observed that following treatment with gemcitabine, a patient who had advanced urothelial carcinoma exhibited disease progression. The patient experienced long-term complete remission after receiving only a domestic PD-1 inhibitor (sintilimab) (12). Fukuta et al. also described a case of advanced urothelial carcinoma that progressed following adjuvant treatment with gemcitabine and carboplatin. Likewise, the tumor reached complete remission after ten cycles of a PD-1 inhibitor (pembrolizumab) alone (13).

Although tislelizumab has achieved good efficacy in the treatment of various malignant tumors, medicine adverse reactions have also occurred. Ye et al. reported that 106 out of 113 patients (94%) experienced at least 1 adverse event (AE) which was related to tislelizumab in a single-arm phase 2 study. The most prevalent treatment-related AEs (TRAEs) were anemia (n=31; 27%) and pyrexia (n=22; 20%). A total of 31 (27%) patients experienced immune-related adverse events (irAEs). IrAEs occurring in ≥5% of patients were skin adverse reactions (n=13; 12%), hypothyroidism (n=12; 11%), and hyperthyroidism (n=7; 6%) (6). Shen et al. reported that most TRAEs were Grade 1 or 2, with the most common being anemia (n=70; 23%). Among all patients, the most common irAEs were increased aspartate aminotransferase (n=59; 20%) and increased alanine aminotransferase (n=54; 18%) (7). In another study, the mostly common TRAEs were fatigue (28%), nausea (25%) and decreased appetite (20%), and anemia (4.9%) was the most common grade 3–4 AE. Potential irAEs occurring in ≥5% of patients were rash (14.6%), diarrhea (6.9%) and hypothyroidism (6.0%) (14).

The case reported here exhibited mild side effects of the skin throughout therapy, with scattered gray papules appearing on the skin of the patient’s limb. The AE was grade 1 severity (rash covering <10% body surface area) according to common terminology criteria for adverse events (CTCAE). It is recommended to continue ICIS therapy, treat the patients with topical emollients and/or mild-moderate potency topical corticosteroids and counsel patients to avoid skin irritants according to ASCO (American Society of Clinical Oncology) guideline (15). The patient had only minor adverse skin reactions, so we did not stop using the ICIs, nor did we use any medication, and the papules on the skin resolved on their own after two weeks. Many mechanism-based toxicities, known as rAEs hinder the process of employing ICIs to treat cancer (4). There have been many reports about irAEs, which can damage the majority of the body’s tissues and organs, including the adrenal glands, heart, liver, pituitary, lungs and kidneys (16–19). The mechanism of irAEs is yet unknown. It is usually thought to be associated with activation of the immune system induced by ICIs and the inflammatory response of many organs throughout the body (20). IrAEs can affect practically every tissue or organ in the body. However, they are more prevalent in single organs, and irAEs involving multiple organs are uncommon (18). According to reports, among the patients treated with PD-1/PD-L1 inhibitors, 60%-70% of them can experience damage to a single target organ (21).

There is no obvious biomarker that can predict the efficacy of PD-1/PD-L1 inhibitors, and the link between the expression level of PD-L1 and the treatment response to PD-1/PD-L1 inhibitors is still unknown. A randomized phase III trial (KEYNOTE-045) showed that regardless of the expression level of PD-L1, there was no significant difference in the survival benefit among the population (22). Chang et al. discovered, by a meta-analysis of 1251 patients, that high expression of PD1 and PDL1 was associated with a poor result in advanced lung cancer but a good outcome in early lung cancer. As a result, they believed that the expression levels of PD1 and PDL1 are possible factors for determining the prognosis of patients with lung cancer (23). Chen et al. discovered that compared to the negative group, PD-L1-positive patients had a longer overall survival (hazard ratio=0.34) and greater objective response rate (relative risk=1.98) and progression-free survival (hazard ratio=0.61), indicating that PD-L1-positive patients may obtain better therapeutic effects from anti-PD-1/PD-L1 therapy. As a result, they stated the expression level of PD-L1 may be a suitable biomarker for predicting the prognosis of patients with gynecological cancer (24). However, more studies are needed to confirm the link between the expression level of PD-L1 and the therapeutic response to PD-1/PD-L1 inhibitors. We did not detect the expression level of PD-L1 in the case we reported because we did not obtain the patient’s tumor specimens.

The current patient has attained only partial remission. Many studies have reported that the combined use of tislelizumab with other chemotherapeutic drugs can achieve complete remission in a variety of advanced tumors. Ding et al. described a case of low-dose decitabine coupled with tislelizumab effectively treating refractory Hodgkin lymphoma that had failed after eight regimens, including PD-1 blockade, multicycle chemotherapy, anti-CD47 antibody therapy, and so on (25). Yao et al. reported a case of tislelizumab coupled with azacitidine achieving full remission in a patient with relapsed acute myeloid leukemia (AML) (26). We will continue to use immunotherapy alone to determine whether complete remission is possible. If the patient and family members agree, we will consider whether we can increase the chemotherapy regimen (such as using gemcitabine) and hope that complete remission can be achieved.

We believe that our study provides a good treatment strategy for patients with isolated renal urothelial cancer who are unwilling to undergo surgery or chemotherapy. There will be more effective and accurate predictive biomarkers to predict which patients will benefit from immunotherapy in the future. In addition, more immunosuppressants will be developed for the treatment of different tumors.

However, our work has some limitations. Firstly, the patient was diagnosed with carcinoma of the left renal calyx based on the patient’s previous history of urothelial carcinoma, urine cytology and CT results, and no tumor tissue was obtained for the pathological investigation. Secondly, tislelizumab was approved for patients with advanced or metastatic urothelial cancer who have previously received platinum-based treatment (6). The patient was not diagnosed with advanced or metastatic urothelial carcinoma and had not received platinum-based chemotherapy. Therefore, the case was not within the indications of tislelizumab. The patient refused surgery and chemotherapy and signed a written informed consent form to participate in the study. Thirdly, the expression level of PD-L1 was not detected because the tissue of the carcinoma was not obtained.

In conclusion, PD-1 inhibitors alone may be an effective treatment strategy for isolated renal calyceal urothelial carcinoma, and more data from research are needed to evaluate whether this treatment is safe and effective.

## Data Availability Statement

The raw data supporting the conclusions of this article will be made available by the authors, without undue reservation.

## Ethics Statement

The studies involving human participants were reviewed and approved by the Human Ethics Committee of Affiliated Hospital of Guangdong Medical University. The patients/participants provided their written informed consent to participate in this study. Written informed consent was obtained from the individual(s) for the publication of any potentially identifiable images or data included in this article.

## Author Contributions

HL, JL and ZX treated the patient. SL, HL and YZ collected data and organized pictures. SL wrote the manuscript. HL revised the manuscript. All authors contributed to the article and approved the submitted version.

## Funding

This work was financially supported by the Natural Science Foundation of Guangdong Province (2019A1515011454), Zhanjiang Science and Technology Plan Project (2019A01028, 2020A01022) and Research Foundation for Advanced Talents of Affiliated Hospital of Guangdong Medical University (20401Z20190003).

## Conflict of Interest

The authors declare that the research was conducted in the absence of any commercial or financial relationships that could be construed as a potential conflict of interest.

## Publisher’s Note

All claims expressed in this article are solely those of the authors and do not necessarily represent those of their affiliated organizations, or those of the publisher, the editors and the reviewers. Any product that may be evaluated in this article, or claim that may be made by its manufacturer, is not guaranteed or endorsed by the publisher.
