# Supplementary material for: Isolated Renal Calyceal Urothelial Carcinoma Effectively Treated With PD-1 Inhibitor Alone: A Case Report And Literature Review
Source: Front Oncol. 2022 May 10;12:866013. doi: 10.3389/fonc.2022.866013 (PMC9128378; doi:10.3389/fonc.2022.866013)
Supplement: Supplementary file 1 [file DataSheet_1.pdf]

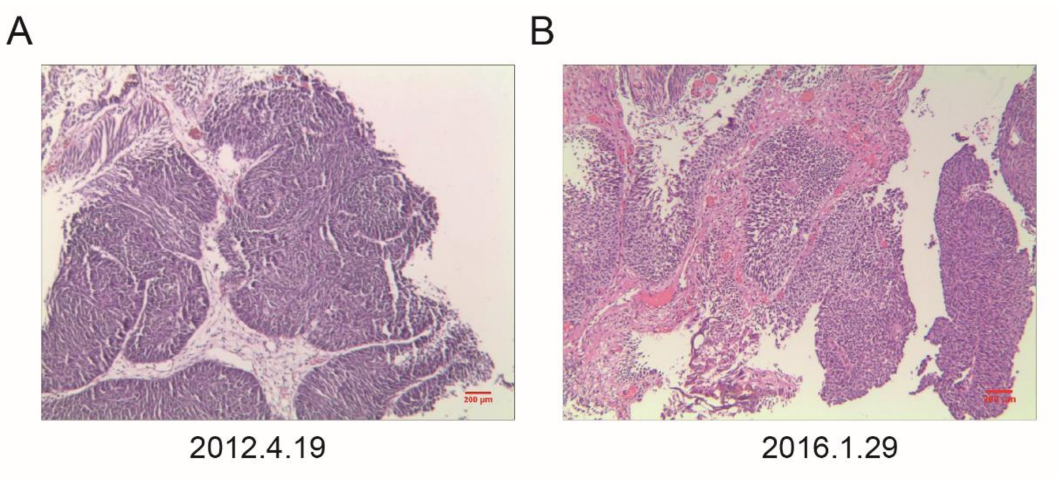

Supplementary Figure 1 Pathological results of the bladder tissues removed from the two operations in 2012 and 2016. (**A**) Pathological results showed low-grade noninvasive papillary urothelial cancer. (**B**) Pathological results showed that bladder urothelial carcinoma had penetrated into the lamina propria.

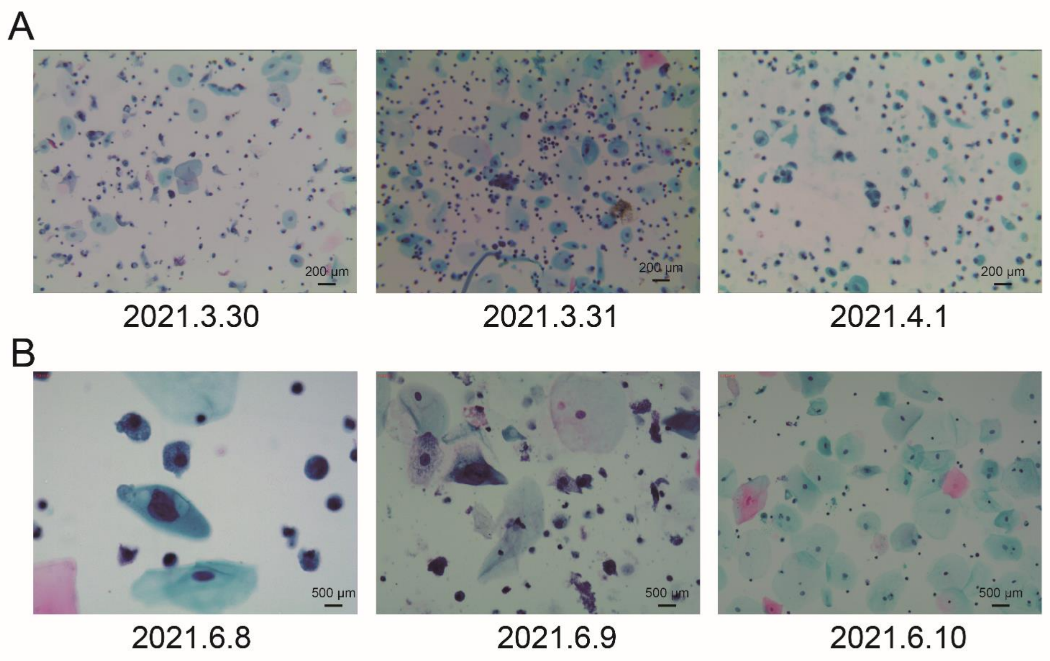

Supplementary Figure 2 Urine cytology before and after immunotherapy. **(A)** Before immunotherapy. Three urine cytology results showed a propensity for low-grade atypical urothelial cells. **(B)** After the fourth immunotherapy. Two urine cytology results revealed atypical urothelial cells, and one urine cytology result was negative.
